# Pituitary T1 signal intensity at magnetic resonance imaging is reduced in patients with obesity: results from the CHIASM study

**DOI:** 10.1038/s41366-023-01338-w

**Published:** 2023-07-21

**Authors:** Giulia Puliani, Emilia Sbardella, Alessia Cozzolino, Valentina Sada, Rossella Tozzi, Chiara Andreoli, Marco Fiorelli, Claudio Di Biasi, Diletta Corallino, Andrea Balla, Alessandro M. Paganini, Mary Anna Venneri, Andrea Lenzi, Carla Lubrano, Andrea M. Isidori

**Affiliations:** 1 Oncological Endocrinology Unit, Regina Elena National Cancer Institute IRCCS, Via Elio Chianesi 53, 00144 Rome, Italy; 2 Department of Experimental Medicine, Sapienza University of Rome, Viale Regina Elena 324, 00161 Rome, Italy; 3 Unit of Emergency Radiology, Department of Radiological, Oncological and Pathological Sciences, Umberto I University Hospital, Sapienza University of Rome, Viale del Policlinico 155, 00161 Rome, Italy; 4 Department of Human Neurosciences, Sapienza University of Rome, Viale dell’Università, 30, 00185 Rome, Italy; 5 Bariatric Surgery Unit, Department of General Surgery and Surgical Specialties “Paride Stefanini” Sapienza University of Rome, Umberto I University Hospital, Viale del Policlinico 155, 00161 Rome, Italy

**Keywords:** Obesity, Clinical trials

## Abstract

**Background:**

Despite obesity being well known to be associated with several pituitary hormone imbalances, pituitary appearance in magnetic resonance imaging (MRI) in patients with obesity is understudied.

**Objective:**

To evaluate the pituitary volume and signal intensity at MRI in patients with obesity.

**Methods:**

This is a prospective study performed in an endocrine Italian referral center (ClinicalTrial.gov Identifier: NCT03458533). Sixty-nine patients with obesity (BMI > 30 kg/m^2^) and twenty-five subjects without obesity were enrolled. Thirty-three patients with obesity were re-evaluated after 3 years of diet and lifestyle changes, of whom 17 (51.5%) achieved a > 5% loss of their initial body weight, whereas the remaining 16 (48.5%) had maintained or gained weight. Evaluations included metabolic and hormone assessments, DEXA scan, and pituitary MRI. Pituitary signal intensity was quantified by measuring the pixel density using ImageJ software.

**Results:**

At baseline, no difference in pituitary volume was observed between the obese and non-obese cohorts. At the 3-year follow-up, pituitary volume was significantly reduced (*p* = 0.011) only in participants with stable-increased body weight. Furthermore, a significant difference was noted in the mean pituitary intensity of T1-weighted plain and contrast-enhanced sequences between the obese and non-obese cohorts at baseline (*p* = 0.006; *p* = 0.002), and a significant decrease in signal intensity was observed in the subgroup of participants who had not lost weight (*p* = 0.012; *p* = 0.017). Insulin-like growth factor-1 levels, following correction for BMI, were correlated with pituitary volume (*p* = 0.001) and intensity (*p* = 0.049), whereas morning cortisol levels were correlated with pituitary intensity (*p* = 0.007). The T1-weighted pituitary intensity was negatively correlated with truncal fat (*p* = 0.006) and fibrinogen (*p* = 0.018).

**Conclusions:**

The CHIASM study describes a quantitative reduction in pituitary intensity in T1-weighted sequences in patients with obesity. These alterations could be explained by changes in the pituitary stromal tissue, correlated with low-grade inflammation.

## Introduction

Obesity is a metabolic disease that has reached epidemic proportions: the World Health Organization (WHO) has declared it the largest global chronic health problem in adults [1]. In 2016, over 650 million adults were obese [2]. It is estimated that by 2030, the prevalence of severe obesity will have doubled compared with that in 2018, reaching 11% [3]. Obesity is well known to be associated with cardiovascular, orthopedic, reproductive, and metabolic complications, with a high economic impact [4]. Several endocrinological alterations have been described in patients with obesity, including a decrease in growth hormone (GH) and insulin-like growth factor (IGF-1) levels [5, 6], hypogonadism [7], and an increase in thyroid-stimulating hormone (TSH) levels [8]. All hypothalamic–pituitary axes can be affected by obesity [9]. Despite this, the pituitary morphology in such patients is understudied. Recent studies have demonstrated various pituitary defects in these patients, including the hypoplastic gland, globular posterior gland, abnormal posterior pituitary gland position, and complete absence of the posterior pituitary gland [10]. Moreover, the pituitary volume may be altered, with some authors reporting a higher prevalence of empty sella [11] and others an increased pituitary volume in patients with obesity [12].

This study primarily aimed to evaluate pituitary volume and signal intensity at magnetic resonance imaging (MRI) in patients with obesity at baseline and after a 3-year follow-up, stratifying patients by the degree of weight change they had achieved. The secondary aim was to assess new quantitative morphological parameters for the study of the sellar region and correlate them with obesity-associated detrimental hormone alterations.

## Materials and methods

### Study design

The CHIASM (Changes in the hypothalamic–pituitary region of patients with metabolic syndrome and obesity) study was designed as a single-center longitudinal prospective observational study of patients with obesity followed in our endocrinology outpatient clinic (Rome). The study was registered in ClinicalTrials.gov (NCT 03458533).

The following were the inclusion criteria: age between 18 and 70 years and body mass index (BMI) higher than 30 kg/m^2^ (obese cohort). The following were the exclusion criteria: overt endocrinopathies (e.g., acromegaly and Cushing’s syndrome), active malignant neoplasia, current use of psychotropic drugs, renal failure, pituitary mass, contraindications to MRI, active infectious disease, use of anti-inflammatory drugs, and chronic inflammatory disease.

Patients with obesity who met the inclusion criteria were enrolled between December 2016 and December 2019. Of the initial 81 consecutive patients with obesity, 5 were excluded, and 7 refused to participate; therefore, a total of 69 participants were enrolled. Moreover, they were compared with a cohort of 25 age- and sex-matched participants without obesity, who had undergone pituitary MRI and hormone evaluations during the same period for suspected pituitary disease, which was subsequently excluded. The study followed an approximate 2:1 recruitment pattern. All participants underwent regular follow-up as clinically indicated, with a routine clinical examination, and the obese cohort underwent a repeat MRI scan 30–36 months after enrollment. For ethical reasons, the non-obese cohort did not undergo a 3-year follow-up MRI scan (no clinical indication to undergo pituitary MRI).

**Fig. 1:**
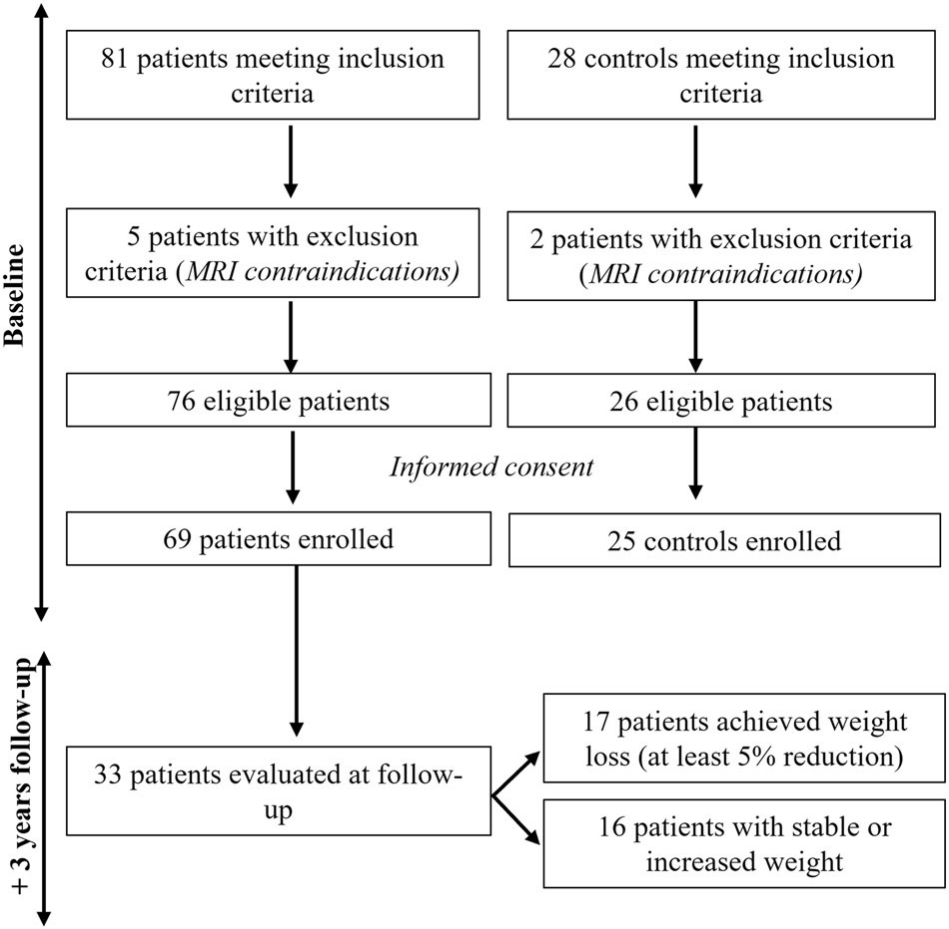
Study design. The flowchart summarizes the number of patients enrolled and the subgroups division.

Patients with obesity were prescribed a personalized diet and other lifestyle modifications, in accordance with good clinical practice. We prescribed a personalized diet to all patients, following the principle of a Mediterranean diet [13, 14]. Additionally, an increase in physical activity was suggested to all patients, including a 40-min walk without interruption three times a week. The target weight loss was defined as a stable weight reduction of at least 5% for at least 12 months before the follow-up MRI scan. Thirty-three participants underwent a second MRI at the study end. They were divided into subgroups of individuals who had achieved stable weight loss and individuals who had not (Fig. 1).

This study was conducted in accordance with the Declaration of Helsinki and was approved by the Ethics Committee of Sapienza, University of Rome (reference number: 4249). All participants provided written informed consent. The study adhered to the Strengthening the Reporting of Observational Studies in Epidemiology (STROBE) guidelines.

### Outcomes

The primary objective of the study was to evaluate pituitary volume at baseline and after a 3-year follow-up. The secondary objectives were the identification and characterization of new morphological and quantitative magnetic imaging parameters of the pituitary gland and the correlations between these parameters and pituitary axis function, body composition, and glycometabolic parameters at baseline and after diet and lifestyle interventions.

### Procedures

The following data were collected for all participants: age, sex, BMI, waist, and hip circumference, drugs, and comorbidities. Blood samples were taken from all participants for biochemical examination, including pituitary hormone evaluation (see also Supplementary). All participants underwent an overnight dexamethasone suppression test to exclude Cushing’s syndrome. The same hormone assay (performed in the laboratory of Umberto I University Hospital, Rome) was used for all examinations. The range of normality for IGF-1 was age and sex-dependent. Body composition was evaluated using the DEXA scan (Horizon® DXA System, Hologic); the visceral fat was estimated using the truncal fat.

**Fig. 2:**
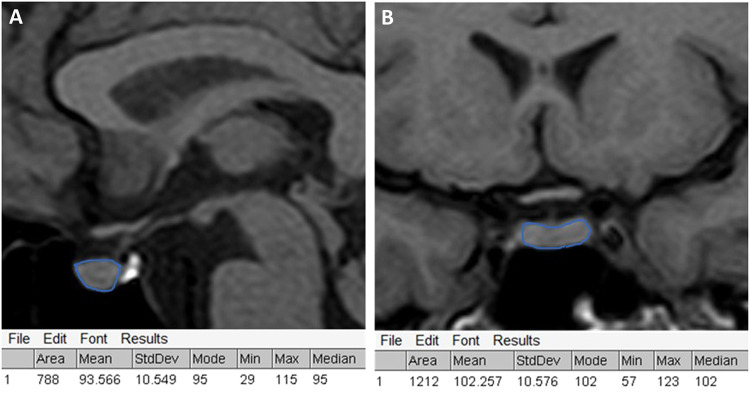
MRI image analysis. Example of marking the region of interest in coronal (**A**) and sagittal (**B**) pituitary RM scans and results of the image analysis using ImageJ software.

All participants underwent contrast-enhanced pituitary MRI (protocol available in Supplementary). Pituitary gland signal intensity was quantified by recording pixel density and distribution in all sequences using ImageJ® software. Two operators independently placed the region of interest (ROI) to entirely cover the pituitary gland, excluding the posterior pituitary bright spot, calculating the mean intensity and its standard deviation (Fig. 2). To obtain the coronal and sagittal intensity, ROIs have been drawn on images containing the pituitary parenchyma, and the mean value has been used for the analysis. The intensity of the white brain matter with normal appearance was used for normalization. Pituitary volume (PV) was calculated using the ellipsoid formula (height × width × depth × π/6).

### Statistical analysis

The statistical analysis was performed using Statistical Package for the Social Sciences (version 20, IBM, Armonk, NY, USA). Values were expressed as means ± standard deviations unless otherwise stated. The normality of the distribution of variables was assessed using the Shapiro–Wilk test (*p* > 0.05). Log transformation or reciprocal transformation was used to correct for skewed data. Pearson’s correlation coefficient was used to assess all correlations. The difference between the binomial proportions of two independent groups on a dichotomous dependent variable was assessed using the chi-square test for homogeneity. A comparison of data between groups was performed using the *t*-test for unpaired samples. *P* < 0.05 was considered statistically significant. Differences between groups in the change from baseline to the 3-year follow-up were evaluated using an analysis of covariance (ANCOVA) model that included baseline outcome as a covariate and weight loss as a fixed effect. Differences between cohorts at baseline were evaluated using an ANCOVA model that included age and white matter as covariates and cohorts as a fixed effect. In the post-hoc comparisons, Bonferroni correction was applied to the *p* values when appropriate, multiplying the raw *p* values corresponding to each statistical test for the number of comparisons to keep the threshold for significance set at *p* < 0.05 [15].

### Sample size calculation

The primary efficacy variable was the difference in the pituitary volume between the subgroups (participants with stable or increased weight and with weight loss) at follow-up. The null hypothesis was no difference in the pituitary volume noted between the two subgroups. A significance level of 0.05 was required for the rejection of the null hypothesis. At the time the study was planned, the only study reporting follow-up data in a healthy population described a PV of approximately 719 ± 159 mm^3^, which was stable after 2.7 years of follow-up (688 ± 164 mm^3^) [16]. The same study also reported a PV change in patients with schizophrenia, from a baseline of 768 ± 125 mm^3^ to 830 ± 134 mm^3^ at follow-up [16]. On this basis, considering the absence of population-based studies on PV in patients with obesity, we calculated a sample size of 14 participants for each subgroup (weight loss vs. stable/increased weight) to achieve an 80% power to detect a difference between the two subgroups at follow-up with a significance level (alpha) of 0.05 using a two-sided one-sample *t*-test. Estimating that approximately only one-third of the participants would achieve a stable 5% weight reduction at follow-up and that an additional third would drop out at follow-up, a minimum sample size of 65 was considered necessary to complete the study.

## Results

### Study population

A total of 69 patients with obesity (16 men [23.19%] and 53 women [76.81%]) were enrolled in this prospective observational study. The mean age was 45.28 ± 12.33 years. Based on BMI, 21, 16, and 32 participants had grades 1 (30–35 kg/m^2^), 2 (35–40 kg/m^2^), and 3 (>40 kg/m^2^) obesity, respectively. Patients with obesity were compared with a cohort of 25 age- and sex-matched patients without obesity. As expected, a significant difference (*p* < 0.001) in weight, BMI, waist, and hip circumference, total fat mass, lean mass, and truncal fat mass was observed between the two cohorts, as summarized in Table 1.

**Table 1:** Study population characteristics.

	Obese cohort (BMI ≥ 30 kg/m^2^)	Non-obese cohort (BMI < 30 kg/m^2^)	*p*
*N*	69	25	
Age (years)	45.28 ± 12.33	43.36 ± 14.68	0.529
Sex			0.935
Men, n/total (%)	16/69 (23.19%)	6/25 (24.00%)	
Women, n/total (%)	53/69 (76.81%)	19/25 (76.00%)	
Anthropometric parameters			
Weight	106.1 ± 22.86	75.26 ± 7.54	**<0.001**
BMI	38.76 ± 6.41	26.00 ± 3.26	**<0.001**
Waist circumference	118.4 ± 14.41	96.91 ± 11.09	**<0.001**
Hip circumference	122.9 ± 12.54	103.9 ± 3.44	**<0.001**
Pituitary MRI			
Pituitary volume (mm^3^)	403.2 ± 159.7	404.2 ± 157.6	0.979
Total empty sella, n/total (%)	4/69 (5.79%)	0/25 (0%)	0.212
DEXA evaluation			
Total fat, %	38.02 ± 6.67	30.72 ± 7.79	**<0.001**
Total fat, kg	39.50 ± 11.85	22.45 ± 6.94	**<0.001**
Lean mass, %	60.95 ± 7.17	68.62 ± 7.47	**<0.001**
Lean mass, kg	60.98 ± 11.96	48.47 ± 8.11	**<0.001**
Truncal fat, %	36.98 ± 6.42	27.66 ± 8.36	**<0.001**
Truncal fat, g	18.81 ± 7.05	9.81 ± 3.96	**<0.001**
Biochemical assessment			
Glucose	101.2 ± 31.78	90.59 ± 9.46	0.105
Insulin	21.71 ± 20.17	9.49 ± 10.66	**0.028**
HOMA-index	5.61 ± 6.23	2.16 ± 2.59	**0.001**
HbA1c	6.23 ± 4.34	5.16 ± 0.46	0.240
Urate	5.38 ± 1.33	4.66 ± 0.94	**0.018**
White blood cells	7.65 ± 1.99	6.37 ± 1.77	**0.006**
Neutrophils	4.25 ± 1.52	3.13 ± 1.47	**0.020**
Lymphocytes	2.23 ± 0.89	1.72 ± 0.71	**0.005**
Neutrophil-to-lymphocyte ratio	2.05 ± 0.80	1.86 ± 0.56	0.281
ESR	25.17 ± 18.17	15.63 ± 9.91	**0.030**
C-reactive protein	1.07 ± 2.25	0.36 ± 0.37	0.140
Fibrinogen	352.6 ± 109.4	285.7 ± 80.90	**0.009**
Hormone evaluation			
TSH	2.02 ± 1.45	2.23 ± 1.76	0.550
FT3	3.27 ± 0.85	3.01 ± 0.70	0.169
FT4	1.23 ± 0.35	1.45 ± 1.81	0.554
PRL	22.21 ± 42.95	22.22 ± 42.95	0.447
GH	0.94 ± 1.63	1.71 ± 2.61	0.201
IGF-1 (ng/L)	158.6 ± 70.7	155.5 ± 78.72	0.894
ACTH (pg/mL)	34.38 ± 16.60	40.73 ± 24.54	0.322
Cortisol (nmol/L)	364.7 ± 126.1	364.3 ± 162.5	0.730
Testosterone (men) (ng/dL)	4.00 ± 1.88	4.45 ± 2.33	0.655
Vitamin D < 30 ng/mL or replacement	52/69 (75.36%)	17/25 (68.00%)	0.710

Variables showing a statistically significant difference between the cohorts are shown in bold.

Thirty-three participants underwent a follow-up examination. They were subsequently divided into subgroups of 17 participants (51.5%) who had lost ≥5% of their initial body weight and 16 (48.5%) who had not. At baseline, all patients with obesity received a personalized diet and suggestions for increasing physical activity. They were periodically re-evaluated to assess the results of diet and lifestyle changes and modify the diet if appropriate.

### Primary outcomes: PV

The mean PV was 403.2 ± 159.7 and 404.2 ± 157.6 mm^3^ in the obese and non-obese cohorts, respectively, with a non-significant difference (*p* = 0.979). However, a negative correlation was observed between BMI and PV in the cohort of patients with obesity (*p* = 0.049) although not in the non-obese cohort (*p* = 0.927).

A total empty sella, defined as a pituitary height of <2 mm, was noted in four patients with obesity and none in those without obesity; however, this difference was not statistically significant (*p* = 0.212). A partial empty sella, defined as a pituitary height between 2 and 5 mm, was noted in 28 of 69 patients with obesity (41%) and 10 of 25 patients without obesity (40%) (*p* = 0.768).

During follow-up evaluation, no change was observed in the mean PV from the baseline in the subgroup who had lost 5% of their initial body weight (from 371.6 ± 132.8 to 367.8 ± 131.7 mm^3^; *p* = 0.868), whereas a significant reduction was observed in the mean PV in the subgroup with stable or increased weight (from 349.8 ± 140.9 to 290.7 ± 128.3 mm^3^; *p* = 0.011). Moreover, two patients with obesity with normal pituitary height, neither of whom had lost any weight over the study period, fell into the partial empty sella category at the 3-year follow-up. ANCOVA controlling for age and baseline volume confirmed that a significant difference was observed in the mean pituitary-volume changes between the subgroups (Table 2). Accordingly, an inverse correlation was noted between the weight loss (in kg) and the PV variation after correction for basal volume (*p* = 0.047). No correlations were noted between the baseline PV and changes in body weight.

**Table 2:** Difference in pituitary MRI volume and signal intensity changes between the subgroup who achieved weight loss and the subgroup with stable or increased weight at follow-up.

	Subgroup with stable weight loss (decrease from baseline > 5%)	Subgroup with stable or increased weight	*p*
*N*	17	16	
Volume change	−0.40 (−41.6 to 40.7) mm^3^	−63.3 (−108.8 to −17.9) mm^3^	0.047*
BMI change	−6.64 ± 5.68 kg/m^2^	2.02 ± 2.17 kg/m^2^	**<0.001**
Truncal fat change	−4.20 ± 6.38	3.05 ± 4.11	**0.003**
T1-weighted signal change	−0.17 (−4.26 to 3.92) %	−6.60 (−10.69 to −2.50) %	0.012**
T1-weighted post-enhanced signal change	2.25 (−4.58 to 9.07) %	−11.49 (−19.25 to −3.73) %	0.017**

Variables showing a statistically significant difference between the cohorts are shown in bold.

*Values are expressed as estimated means (lower-upper limit of 95% confidence interval [CI]). Covariates in the model: age and baseline volume.

**Values are expressed as estimated means (lower-upper limit of 95% CI). Covariates in the model: age, baseline, and 3-year follow-up white matter signal intensity.

### Pituitary intensity

In basal T1-weighted images, the pituitary intensity index in patients with obesity was reduced compared with those without obesity (sagittal: 80.13 ± 18.08 vs. 84.26 ± 17.16, *p* = 0.043; coronal: 75.10 ± 14.27 vs. 82.66 ± 13.90, *p* = 0.028). Post-enhanced MRI scans confirmed the difference in signal intensity between the two cohorts (sagittal: 118.53 ± 19.05 vs. 136.27 ± 27.36, *p* = 0.003; coronal: 129.98 ± 28.50 vs. 141.52 ± 19.46, *p* = 0.003). No difference in pituitary signal intensity in T2-weighted images was observed between the cohorts. ANCOVA controlling for age and white matter intensity confirmed that the obese cohort had a significantly lower mean pituitary intensity (Table 3).

**Table 3:** Difference in T1-weighted basal and contrast-enhanced mean pituitary intensity index between the cohorts (ANCOVA).

	Obese cohort	Non-obese cohort	*p*
Mean basal coronal pituitary intensity (T1)	75.439 (73.134–77.744)	81.744 (77.918–85.572)	**0.006**
Mean basal sagittal pituitary intensity (T1)	77.597 (74.214–80.979)	84.876 (79.480–90.271)	**0.026**
Mean contrast-enhanced coronal pituitary intensity (T1)	129.309 (124.176–134.442)	145.503 (136.896–154.109)	**0.002**
Mean contrast-enhanced sagittal pituitary intensity (T1)	118.107 (113.023–123.190)	137.516 (128.762–146.270)	**0.001**

Values are expressed as estimated means (lower-upper limit of 95% CI). Covariates in the model: age and white matter signal intensity. Variables showing a statistically significant difference between the cohorts are shown in bold.

When participants were subgrouped according to the grade of obesity (BMI 30–35 kg/m^2^ vs. BMI > 40 kg/m^2^), a higher BMI was associated with a more marked reduction in the mean coronal post-enhanced T1-weighted pituitary intensity (*p* = 0.005).

At the 3-year follow-up, T1 pituitary intensity was unchanged in the subgroup of patients with obesity who had lost weight; however, it was significantly reduced in both basal and post-enhanced scans in the subgroup with stable or increased body weight, with a significant difference between the subgroups (basal, *p* = 0.012; post-enhanced, *p* = 0.017) (Table 2). Accordingly, following correction for the pituitary intensity of the same sequences at baseline, a negative correlation was noted between weight loss and T1-weighted coronal and sagittal pituitary intensities and post-enhanced T1-weighted sagittal pituitary intensity (*p* < 0.001; *p* =  0.005; and *p* < 0.001, respectively). No correlations were observed between the baseline pituitary intensity and changes in body weight.

### Determinants of pituitary intensity

**Fig. 3:**
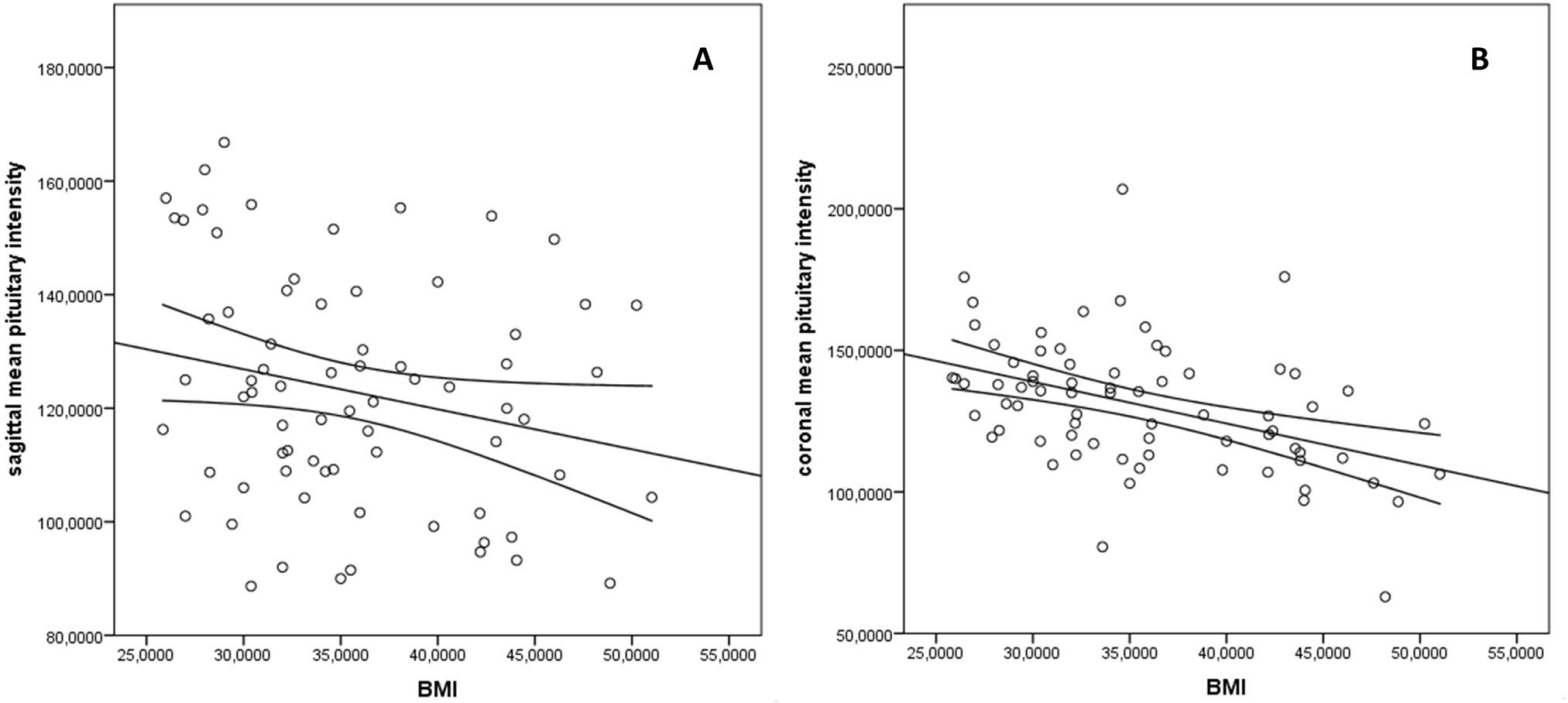
Correlation analyses. **A** Scatter plot showing the correlation between mean pituitary intensity in T1-weighted post-enhanced sagittal scan and body mass index (BMI) (R^2^ linear 0.231). **B** Scatter plot showing the correlation between mean pituitary intensity in T1-weighted post-enhanced coronal scan and BMI (R^2^ linear 0.231).

After controlling for age and white matter signal intensity, a negative correlation was noted between post-enhanced T1-weighted pituitary intensity and BMI (sagittal scan, r = −0.265, *p* =  0.020; coronal scan, r = −0.381, *p* < 0.001) (Fig. 3). Body composition studies revealed that pituitary signal intensity (coronal T1-weighted basal images adjusted for white matter and age) was negatively correlated with the truncal fat (r = 0.325, *p* = 0.006) although not with the total fat mass (*p* = 0.918).

Furthermore, a negative correlation was noted between the pituitary intensity and the inflammatory index. After correction for white matter and age, fibrinogen levels were significantly different between the two subgroups (*p* = 0.009) and was inversely correlated with the mean pituitary intensity, in both basal (sagittal scans, r = −0.225, *p* = 0.042; coronal scans, r = −026, *p* =  0.018) and post-enhanced sequences (sagittal scans, r = −0.366, *p* = 0.002).

After adjusting for white matter intensity, multiple regression analysis showed that the percentage of truncal fat and fibrinogen were significant predictors of the mean intensity of coronal T1-weighted scans (*p* = 0.001). The model explained up to 21% of the variance of the pituitary signal intensity.

An upward change in BMI was negatively correlated with T1-weighted sagittal (r = −0.528, *p* = 0.002), coronal (r = −0480, *p* = 0.006), and post-enhanced sagittal (r = −0.624; *p* =  0.001) pituitary signal intensities. Similarly, an upward change in truncal fat was also negatively correlated with the same parameters (r = −0.563, *p* = 0.003; r = −0.475, *p* = 0.019; and r = −0.552, *p* = 0.008, respectively).

### Hormone and metabolic assessment

No difference was observed in pituitary hormones levels between the two cohorts, whereas metabolic indexes, including the HOMA index, and glycated hemoglobin, were higher in patients with obesity (Table 1). Cortisol levels were directly correlated with pituitary intensity in both basal scan (*p* = 0.006) and post-enhanced sequences (*p* = 0.033). After correction for BMI, IGF-1 levels were directly correlated with PV (*p* = 0.001) and T1-weighted pituitary intensity (*p* = 0.049). At follow-up, a significant reduction was noted in TSH levels and an increase in testosterone levels (in men) in the subgroup of patients with obesity and weight loss (*p* = 0.030). Significantly, the subgroup of patients with obesity and stable or increased body weight had a higher percentage of patients with metabolic syndrome than the subgroup who had lost weight (10/16; 62.5% vs. 4/17; 23.5%, *p* = 0.024).

## Discussion

This is the first study that prospectively investigates pituitary gland volume and signal intensity in patients with obesity and stable weight loss and suggests a causal link between truncal obesity and pituitary MRI appearance. Hypothalamus–pituitary function and adipose tissue are closely linked in crosstalk with GH, thyroid, adrenal, gonadal hormone, and adipokine secretion. A recent meta-analysis has demonstrated a high prevalence of endocrine disorders in patients with obesity [17]. Several studies have investigated the relationship between obesity and pituitary function: it has long been known that patients with obesity can be GH deficient [5] and exhibit a blunted GH response to several stimuli [6, 18]. The visceral fat and basal insulin are predictors of daily GH levels, independent of age, and sex [19]. Moreover, massive weight loss has been shown to restore the dynamics of GH secretion [6]. Men with obesity show low testosterone levels associated with normal gonadotropin values, and it has been shown that hypogonadotropic hypogonadism is restored by weight loss [20]. Multiple factors contribute to secondary hypogonadism, including inflammatory mediators of low chronic inflammation, such as tumor necrosis factor and interleukin 1; insulin resistance; leptin resistance; and reduced kisspeptin secretion [21]. In patients with morbid obesity, TSH levels are two-fold higher than in controls, and a significant decrease in serum TSH levels can be obtained through weight loss [22]; furthermore, FT4 levels are inversely correlated with BMI [23]. In a study of 193 patients with subclinical hypothyroidism, thyroid function seemed to be negatively correlated with BMI [24]. Finally, patients with obesity showed disrupted cortisol circadian rhythm [25]. Despite all these known alterations, pituitary morphology in patients with obesity is understudied.

The CHIASM study was designed to evaluate quantitative parameters at pituitary MRI to identify possible structural alterations that may underlie and preempt morbid obesity-associated pituitary hormone imbalances. In the present study, to enable the identification of any direct causal effects of excess weight on the pituitary gland, the presence of obesity-induced common endocrine alterations was an exclusion criterion.

No difference was noted in baseline PV between the obese and non-obese cohorts; however, a negative correlation between PV and BMI was observed within the obese cohort only. The mean PVs observed in our cohorts were consistent with the recently published normative data for PV in adults (50–69 years old) [26], confirming that the aim of enrolling normal participants was met.

At the 3-year follow-up, participants who had achieved a stable reduction in body weight showed no changes in PV, whereas those who had stable or increased body weight presented a statistically significant reduction in PV. We decided to use the 5% cut-off of weight loss since it seems the lowest cut-off capable for improving obesity-associated metabolic complications, thereby reducing cardiovascular risk [27]. No difference was noted between the cohorts in the proportion of participants with an empty sella, and the frequency of total empty sella was similar to the reported incidence of 5.5–12% [28, 29]. The indirect correlation between PV and weight loss and the decrease in PV due to obesity persistence is in accordance with published data, which demonstrate that early-onset morbid obesity is associated with the hypoplastic pituitary gland, globular posterior gland, abnormal position of the posterior pituitary, and complete absence of the posterior pituitary gland [10]. In a recent cross-sectional study that used an optimized protocol of high-resolution functional MRI [30], obesity was associated with a reduced hypothalamic volume, highlighting the structural, and functional hypothalamic dysregulation considered to be involved in the development and progression of obesity [31]. Moreover, it has been demonstrated that radiotherapy-induced hypothalamic damage results in volume reduction and metabolic complication development in patients with craniopharyngiomas [32].

To further investigate the pituitary appearance in patients with obesity, we performed the analysis of all MR images using ImageJ® software, a public domain Java image processing and analysis program [33]. This program has also been used in pituitary stalk lesions, wherein the texture analysis of MRI scans revealed a significant correlation between tumor pathology and pituitary stalk heterogeneity in T1-weighted images [34]. Our results showed that the obese cohort had a significantly lower mean pituitary intensity in T1-weighted sequences. Furthermore, this reduction seemed to be correlated with DEXA truncal fat, which is an index of visceral adiposity [35], and less correlated with total fat mass. Therefore; it appears that obesity has a more detrimental effect on pituitary intensity in patients with visceral fat accumulation. Following this interpretation, inflammatory indexes (particularly fibrinogen), which increase in low-grade chronic inflammation owing to visceral obesity [36], were also inversely correlated with the mean pituitary intensity. At the follow-up evaluation, the pituitary intensity had significantly decreased in the subgroup of patients with stable or increased body weight, although not in the subgroup of patients who had achieved significant weight loss through diet and lifestyle changes. This suggests that since obesity-induced pituitary changes are progressive, they can be prevented by dieting and behavioral interventions.

The reduced mean pituitary intensity may be because of a structural change in the pituitary gland. A study on pituitary adenomas demonstrated that the MRI intensity of hormone-secreting adenomas is higher than that of non-secreting adenomas in T1-weighted sequences [37]. Recent studies have correlated the T1-weighted signal with pituitary adenoma consistency [38, 39], and reported that soft adenomas were more hypointense than fibrous adenomas.

The pituitary intensity in T2-weighted sequences has been studied in several pituitary diseases. In acromegaly, T2-hypointense pituitary adenomas show a better hormone response and have greater tumor shrinkage following somatostatin analog therapy [40]. Hyperintensity in T2-weighted images has been demonstrated in patients with obesity and nickel sensitivity compared with nonallergic patients [41]. Hypothalamic T2 hyperintensity has also been associated with an increase in gliosis in obese mice [42], which is a consequence of the inflammatory process that begins promptly after starting a high-fat diet in rodents [43]. It is possible to hypothesize that pituitary MRI hypointensity in patients with obesity is caused by a change in the relative composition of pituitary tissue, with an increase in the stromal portion and a decrease in the endocrine cell portion, in line with the evidence that obesity could also induce hypothalamic gliosis in humans [42].

This study suggests the role of obesity in reducing PV and intensity; however, in contrast, PV, and intensity at baseline do not predict subsequent weight loss. Based on these results, it is possible to hypothesize that pituitary modifications are the consequence of obesity and not the cause.

Interestingly, the present study demonstrated a direct correlation between cortisol levels and pituitary intensity. This correlation could be explained by considering the demonstrated reduction in pituitary intensity due to visceral adiposity. Visceral obesity is associated with an alteration of the hypothalamic–pituitary–adrenal axis characterized by low morning cortisol levels and loss of circadian rhythm [25, 44]. Similarly, Duclos M et al. [45] demonstrated that premenopausal women with obesity with abdominal body fat distribution had lower awakening salivary cortisol levels than those with peripheral fat distribution.

Furthermore, the present study shows a direct correlation between IGF-1 levels and PV, even after correction for BMI, which is consistent with the finding of the role of PV in GH secretion in pediatric populations [46]. As expected, a decrease in TSH levels was observed in the subgroup of patients with weight loss and an increase in testosterone levels at follow-up examination was noted in men.

This study has some limitations. First, less than half of the patients enrolled at baseline completed the final follow-up MRI. One of the causes of the high dropout was the patients’ unwillingness to repeat pituitary MRI. However, a high dropout rate had been anticipated when calculating the sample size, and the pre-determined minimum sample size was achieved. Second, more women were enrolled than men; however, this reflects both the prevalence of obesity and the greater propensity for women to undergo long observational studies and follow dietary weight loss advice. Third, the adherence of the patients to the prescribed diet and physical activity programs was not systematically assessed using questionnaires or diaries; therefore, despite this study aimed to evaluate the effects of body weight change more than diet and lifestyle changes on pituitary MRI parameters, reporting these data is not possible. Fourth, since this study included both a control and a study group and all patients with obesity received a similar diet and lifestyle change suggestion, this study was not randomized. Finally, since this is the first prospective study to quantify pituitary signal intensity in patients with obesity, larger studies or others performed in different populations or in patients with comorbidities, including diabetes mellitus, are needed to confirm, and extend the generalizability of the results.

## Conclusions

This is the first study wherein pituitary intensity in patients with obesity, evaluated using MRI, has been prospectively quantified using specific software rather than merely qualitatively compared with other brain structures. The strength of this post-processing analysis is that the same approach can be applied to archived pituitary MRI scans, using a free, and easily accessible program and without requiring specific acquisition techniques or sequences, thereby allowing comparison with other cohorts. This study demonstrated a decrease in pituitary intensity in patients with obesity, which is linked to visceral adiposity and low-grade chronic inflammation. In patients with obesity who fail to lose weight, both pituitary volume, and intensity could decrease further; however, a 5% decrease in the initial body weight seems sufficient to avoid further pituitary changes. These structural pituitary alterations, likely due to an increase in the gland’s stromal component, may be the cause of the hormonal imbalance typically observed in patients with severe long-standing obesity.

### Supplementary information

Supplementary Materials

## Data Availability

Some or all datasets generated during and/or analyzed during the current study are not publicly available but are available from the corresponding author on reasonable request.
