# Correction: Pituitary T1 signal intensity at magnetic resonance imaging is reduced in patients with obesity: results from the CHIASM study

**DOI:** 10.1038/s41366-023-01363-9

**Published:** 2023-08-28

**Authors:** Giulia Puliani, Emilia Sbardella, Alessia Cozzolino, Valentina Sada, Rossella Tozzi, Chiara Andreoli, Marco Fiorelli, Claudio Di Biasi, Diletta Corallino, Andrea Balla, Alessandro M. Paganini, Mary Anna Venneri, Andrea Lenzi, Carla Lubrano, Andrea M. Isidori

**Affiliations:** 1 Oncological Endocrinology Unit, Regina Elena National Cancer Institute IRCCS, Via Elio Chianesi 53, 00144 Rome, Italy; 2 Department of Experimental Medicine, Sapienza University of Rome, Viale Regina Elena 324, 00161 Rome, Italy; 3 Unit of Emergency Radiology, Department of Radiological, Oncological and Pathological Sciences, Umberto I University Hospital, Sapienza University of Rome, Viale del Policlinico 155, 00161 Rome, Italy; 4 Department of Human Neurosciences, Sapienza University of Rome, Viale dell’Università, 30, 00185 Rome, Italy; 5 Bariatric Surgery Unit, Department of General Surgery and Surgical Specialties “Paride Stefanini” Sapienza University of Rome, Umberto I University Hospital, Viale del Policlinico 155, 00161 Rome, Italy

**Keywords:** Obesity, Clinical trials

Correction to: *International Journal of Obesity* 10.1038/s41366-023-01338-w, published online 21 July 2023

The article was wrongly published under the article type “Review”. Please note that the article is actually a clinical trial and should have therefore been published as an “Original Paper”.

