# Supplementary material for: Pituitary T1 signal intensity at magnetic resonance imaging is reduced in patients with obesity: results from the CHIASM study
Source: Int J Obes (Lond). 2023 Jul 21;47(10):948–55. doi: 10.1038/s41366-023-01338-w (PMC10511316; doi:10.1038/s41366-023-01338-w)
Supplement: Supplementary file 1 — Supplementary Materials [file 41366_2023_1338_MOESM1_ESM.pdf]

SUPPLEMENTARY

Supplementary Methods

RMI protocol

The images were obtained using a head coil on a 1.5-T MR scanner (Siemens® Magnetom Avanto 1,5 Tesla). The imaging protocol included acquisition of pre- and post-contrast-enhanced sagittal and coronal T1-weighted spin-echo (SE) and coronal T2-weighted SE images. The parameters for T1 SE imaging were the following: field of view 180×180; matrix 180×144; 25 slices; slice thickness 3 mm; repetition time (TR) 475 ms; and echo time (TE) 15 ms. The parameters for T2 SE imaging were the following: field of view 180×180; matrix 180×144; 25 slices; slice thickness 3 mm; TR 2900 ms; and TE 120 ms. The post-contrast-enhanced images were obtained an average of three-five minutes after injection of a gadolinium-based contrast agent (Gd-DPTA OmniScan, 15 mL).

Biochemical evaluation

Blood samples were taken from all subjects to assess glucose and lipid metabolism, liver, renal, pancreatic, hematopoietic and coagulative functions, and calcium and phosphate metabolism. Baseline endocrine tests included: serum GH, IGF1, luteinizing hormone (LH), follicle-stimulating hormone (FSH), testosterone (in men) or estradiol (in women), prolactin, thyroid function, 08:00h cortisol, and adrenocorticotropic hormone (ACTH).
